# Contributions of Titin and Collagen to Passive Stress in Muscles from *mdm* Mice with a Small Deletion in Titin’s Molecular Spring

**DOI:** 10.3390/ijms23168858

**Published:** 2022-08-09

**Authors:** Pabodha Hettige, Dhruv Mishra, Henk Granzier, Kiisa Nishikawa, Matthew J. Gage

**Affiliations:** 1 Chemistry Department, University of Massachusetts Lowell, Lowell, MA 01854, USA; 2 Department of Biological Sciences, Northern Arizona University, Flagstaff, AZ 86011-5640, USA; 3 Department of Cellular and Molecular Medicine, University of Arizona, Tucson, AZ 85724, USA

**Keywords:** soleus, fiber bundles, trypsin, KCl, KI, exon splicing, titin extraction

## Abstract

Muscular dystrophy with myositis (*mdm*) is a naturally occurring mutation in the mouse *Ttn* gene that results in higher passive stress in muscle fibers and intact muscles compared to wild-type (WT). The goal of this study was to test whether alternative splicing of titin exons occurs in *mdm* muscles, which contain a small deletion in the N2A-PEVK regions of titin, and to test whether splicing changes are associated with an increase in titin-based passive tension. Although higher levels of collagen have been reported previously in *mdm* muscles, here we demonstrate alternative splicing of titin in *mdm* skeletal muscle fibers. We identified Z-band, PEVK, and C-terminus Mex5 exons as splicing hotspots in *mdm* titin using RNA sequencing data and further reported upregulation in ECM-associated genes. We also treated skinned *mdm* soleus fiber bundles with trypsin, trypsin + KCl, and trypsin + KCL + KI to degrade titin. The results showed that passive stress dropped significantly more after trypsin treatment in *mdm* fibers (11 ± 1.6 mN/mm^2^) than in WT fibers (4.8 ± 1 mN/mm^2^; *p* = 0.0004). The finding that treatment with trypsin reduces titin-based passive tension more in *mdm* than in WT fibers supports the hypothesis that exon splicing leads to the expression of a stiffer and shorter titin isoform in *mdm* fibers. After titin extraction by trypsin + KCl + KI, *mdm* fibers (6.7 ± 1.27 mN/mm^2^) had significantly higher collagen-based passive stress remaining than WT fibers (2.6 ± 1.3 mN/mm^2^; *p* = 0.0014). We conclude that both titin and collagen contribute to higher passive tension of *mdm* muscles.

## 1. Introduction

Titinopathies are striated muscle diseases produced by mutations in the titin gene [1]. Titin was first isolated as an intracellular elastic protein called “connectin” in chicken skeletal muscles [2]. Later, Wang and co-workers coined the term “titin” for the high-molecular-weight elastic protein that was identified via electrophoretic analysis [3]. The *TTN* gene includes 347 exons in mice ([4]; Ensembl transcript version ENSMUST00000099981.9, NCBI Accession: BN001114.1). Due to the colossal size of titin and the presence of alternative splicing sites, titin likely has hundreds of different isoforms [5]. This diversity enables the fine-tuning of healthy muscle function. However, titin-linked diseases also result from mutations that disrupt the native titin protein structure [1].

Titin is the largest (4.2 MDa) and third most abundant protein in striated muscles [6]. With up to 38,000 amino acids [6], the titin protein has an I-band region that is composed of two spring-like elements, the proximal tandem Ig domains and the PEVK region [7]. The N2A segment links the tandem Ig domains to the PEVK region [8]. Titin contributes up to 70% of passive tension in single muscle fibers and intact muscles and up to 98% of passive force in single myofibrils and sarcomeres [9,10,11,12]. Alternative splicing produces different isoforms that result in the tissue specificity and diversity of titin function [13]. The passive tension and the sarcomere length at which passive tension develops are determined by the expression of different titin isoforms [14,15]. For example, extensor digitorum longus (EDL) and psoas fibers express titin isoforms with shorter and stiffer PEVK regions compared to soleus (SOL) fibers that have a longer and more compliant PEVK region [15,16]. Transgenic mice with deletions in titin (IG KO; [17] and Ttn^∆112–158^, [10]) demonstrate increased titin-based passive tension. In the Ttn^∆112–158^ knockout, the higher passive tension is due to the primary deletion, whereas in the small IG knockout, the higher passive tension is due to the deletion of Ig domains and altered splicing of exons in the PEVK region.

The muscular dystrophy with myositis (*mdm*) mutation in mice results in a deletion of 83 amino acids in the N2A and PEVK linker region of titin [18,19]. Studies have shown that the small deletion in *mdm* results in the fibrosis of diaphragm muscles [19,20] and increased passive tension in *mdm* psoas fibers due to the higher collagen content [21]. Powers et al. observed that passive tension did not differ between *mdm* and WT myofibrils from psoas muscles, which supported the hypothesis that the *mdm* mutation has no effect on the titin-based passive tension of myofibrils in which no collagen is present [22]. Other studies found higher passive tension of intact *mdm* SOL and EDL muscles compared to wild-type (WT) [23,24,25]. Despite the small size of the deletion (248 nucleotides and 83 amino acids), which represents a very small proportion of the titin protein, the *mdm* phenotype is complex [19]. *Mdm* mice exhibit abnormal gait [26,27], disordered thermoregulation including shivering [28] and non-shivering thermogenesis [29], stunted growth [27], and premature death [18]. The *mdm* deletion also reduces active muscle stiffness [22,23,30,31].

The primary goal of this study was to test whether alternative splicing of titin exons occurs in *mdm* muscles, which contain a small deletion in the N2A-PEVK regions of titin, because previous studies of transgenic mice with a knockout in this region showed splicing in the PEVK region as well as higher passive tension [17]. We also sought to test whether the large splicing change observed in the PEVK region of soleus muscles was associated with an increase in the titin-based passive tension. We hypothesize that the increased passive tension of *mdm* muscles may be due to alternative splicing of PEVK titin in addition to increased collagen content. To test this hypothesis, we investigated titin exon splicing and other transcriptomic changes using RNA sequencing of *mdm* psoas, soleus, and extensor digitorum longus (EDL) muscles, along with progressive extraction of titin to determine the relative contributions of titin and collagen to passive tension in *mdm* soleus muscles compared to WT. We elected to investigate the passive tension of the soleus muscle since previous studies suggested that its passive tension was more affected by the *mdm* mutation than the EDL [24] or psoas [22].

Wu et al. used progressive trypsin + KCl + KI extraction of titin to investigate the relative contributions of titin and collagen to muscle passive tension [32]. Trypsin is a relatively promiscuous protease that has a high affinity for titin’s PEVK region due to its disordered structure (which assists the trypsin access to the protein) and elevated lysine content [33]. To demonstrate whether trypsin extraction specifically abolishes titin-based passive tension, Wu et al. compared trypsin extraction with KCl + KI treatment, which removes >95% of myosin and actin, titin’s anchors in the sarcomere [32]. When trypsin-treated muscles were then treated with the KCl + KI, no additional reduction in passive tension was observed because titin was already degraded. Thus, trypsin can be used to degrade titin without major effects on other proteins. We predicted that trypsin treatment would reduce passive stress to a greater extent in *mdm* fiber bundles compared to WT if altered exon splicing reduces the PEVK contour length in *mdm*. We also predicted that the higher passive tension would remain after treatment with trypsin + KCl + KI in *mdm* fiber bundles due to the higher collagen content [21].

## 2. Results

### 2.1. RNA Sequencing

The splicing pattern along the titin gene was investigated using an exon-wise percent spliced-in index (PSI) to identify regions with splicing alterations in *mdm* compared to WT muscles. Reference domain annotations and sequence boundaries of titin were extracted from the *Mus musculus* titin GenBank entry BN001114.1 and matched to the Ensembl titin isoform ENSMUST00000099981.9 to locate the corresponding exon coordinates. The Z-disc, PEVK domain, and MEx5 exons were identified as the primary regions contributing to titin length alteration in *mdm* muscles based on the changes in the average exon-wise PSI-indexes, which were calculated using RNA-Seq data. PEVK exons showed an overall decrease in expression, whereas exons located at the Z- and M-lines tended to have higher inclusion rates in *mdm* titin transcripts compared to WT.

#### 2.1.1. Splicing in Z-Repeats

**Figure 1:**
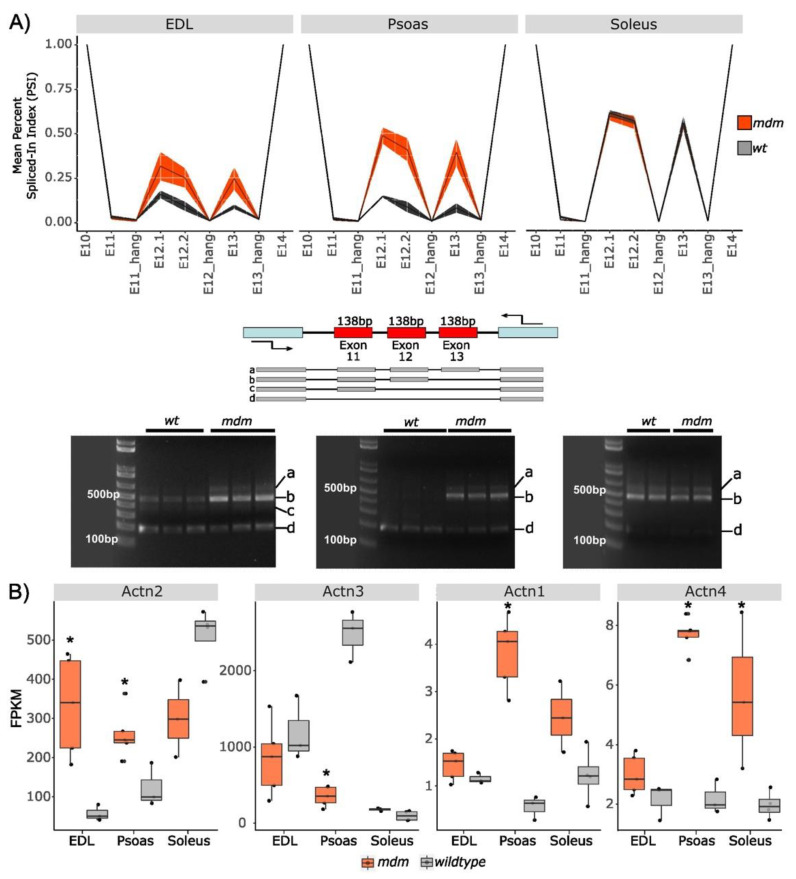
Fast *mdm* muscles (red) expressed more Z-repeats compared to WT muscles (gray). (**A**) Percent spliced-in (PSI) indexes calculated for exons 10–14 using RNA-Seq data (top row), and agarose gel images for amplification of exons 11–13 using primers annealed on flanking exons (bottom row). Expression of exons 12 and 13 encoding Z-repeats 5 and 6 was upregulated in *mdm* EDL and psoas muscles compared to WT muscles (two-way ANOVA, all *p* < 0.0001). PSI index varies from 0 = complete exclusion to 1 = complete inclusion of exons in the transcript pool. Exon labels are suffixed with a period followed by a number if the region was split into separate segments based on the variable distribution of reads in the alignments. Segments flanking annotated exon boundaries are suffixed with “_hang”. The shaded areas show the standard deviation of mean PSI values. Illustration shows the primer locations with arrows and exon arrangement in the region of interest. Agarose gel images show four variants: (a) expression of exons 11–13 (599 bp); (b) exclusion of one exon (461 bp); (c) exclusion of two exons (323 bp); and (d) complete exclusion of exons 11–13 (185 bp). (**B**) Expression of α-actinin1–4 in *mdm* muscles compared to WT muscles. Data were plotted as FPKM (fragments per kilobase of transcript per million mapped reads). * Indicates *mdm* to wild-type expression differences significant at the 99% level as calculated by DESeq2.

In the N-terminus of titin, which coincides with the sarcomere Z-disc and is composed of seven splice-prone Z-repeats (ZR) [34], the expression of ZR 5 and 6 coded by exons 12 and 13 was upregulated in fast muscles (EDL and psoas) with the *mdm* deletion, but Z-repeat expression in *mdm* soleus was unaffected (two-way ANOVA, all *p* < 0.0001; Figure 1A). The observations were supported by PCR experiments in which relatively higher levels of high-molecular-weight amplicons were generated from *mdm* EDL and *mdm* psoas cDNA by flanking primers on ZR 3-7 (exons 10 and 14; Figure 1A). High-intensity bands on the agarose gels indicated the expression of a longer Z-repeat region in *mdm* titin (each exon = 138 bp vs. three exons with flanking regions = 599 bp), which corresponds to the inclusion of coding regions that are generally spliced out in WT titin isoforms. Similar to the RNA-Seq data, no expression differences were observed in the same region in the *mdm* soleus compared to WT muscles.

In *mdm* psoas, three expression variants were amplified in the PCR experiments. The first variant (599 bp) corresponding to the amplification of the complete region of ZR4-6 (exons 11–13), represents a minor band in the transcript pool. The second variant in *mdm* samples corresponding to the exclusion of one Z-repeat within ZR4-6, likely representing the exclusion of ZR4 (461 bp) as seen in the PSI plots, was the dominant band. In the third variant, all the exons encoding ZR4-6 (185 bp) were completely absent. Similarly, all three amplicons observed in the psoas muscle were present in *mdm* EDL, with one additional variant in which two of the Z-repeat exons (323 bp) were absent. Variants missing one Z-repeat within the region and the variant missing all four repeats were the predominant isoforms in WT EDL. Overall, the Z-disc splicing pattern demonstrated an expression of longer isoforms in *mdm* fast muscles compared to their WT counterparts.

In addition, α-actinin-2 (Actn2) expression levels were elevated in the *mdm* fast muscles EDL and psoas (Figure 1B). This observation is consistent with the increased expression of Z-repeats in fast muscles and possibly indicates an increase in the number of titin α-actinin anchoring points at the Z-disc. Actn2 is the primary isoform in oxidative muscle, whereas Actn3 is the predominant isoform in glycolytic muscle [35], so the upregulation of Actn2 in *mdm* muscle might reflect a change in the metabolic pathways in *mdm* muscle. α-actinin-3 (Actn3) was significantly downregulated in the psoas, suggesting a stronger response to *mdm* deletion in the psoas compared to EDL because α-actinin-3 is predominantly expressed in fast muscles. It is interesting to note that although the Z-repeat splicing in *mdm* soleus muscles was not altered, they nevertheless showed increased expression of Actn2 as also observed in the EDL and psoas. The reason for these observations is not immediately obvious.

In addition to examining the two skeletal muscle isoforms of α-actinin (Actn2 and Actn3), we also measured the expression patterns of Actn1 and Actn4, which are the predominant forms in non-muscle tissue [36,37,38,39]. In non-muscle cells, these two isoforms of α-actinin are localized to microfilament bundles that help mediate the membrane attachment of cytoskeletal actin at focal contacts and in actin stress fibers [40,41]. In stress fibers, Actn4 is more commonly localized to circular dorsal ruffles, whereas Actn1 is more evenly distributed throughout the fibers [40,42,43]. Both Actn1 and Actn4 isoforms were upregulated in all three muscles, with the largest change occurring in the psoas muscle. Our previously published transcriptome comparison revealed that psoas muscles have unique gene expression patterns that differ from both EDL and soleus muscles [44], so the transcript differences in the Actn1 and Actn4 genes also appear to be yet another example of the unique expression patterns observed previously in the psoas muscle.

#### 2.1.2. PEVK Splicing

**Figure 2:**
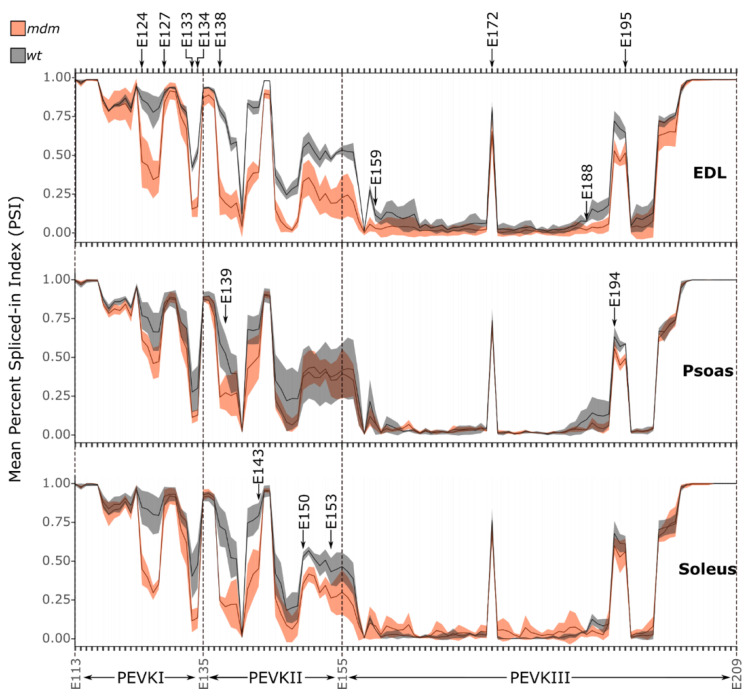
PEVKI and PEVKII exons have significantly lower expression levels in *mdm* muscles. Expression of PEVK exons in *mdm* muscles (red) and WT muscles (grey) varied among regions, with the most variations occurring in PEVKI and PEVKII. PSI index varies from 0 = complete exclusion to 1 = complete inclusion in the transcript pool. The shaded area shows the standard deviation of mean PSI values. Exons undergoing an expression drop in more than one muscle type are shown at the top. Exon regions specific to each muscle are shown in the respective rows. Two-way ANOVA demonstrated significant downregulation of exons 124–127, 133–134, 138–145, and 194 in *mdm* compared to WT, all *p* < 0.0029).

**Figure 3:**
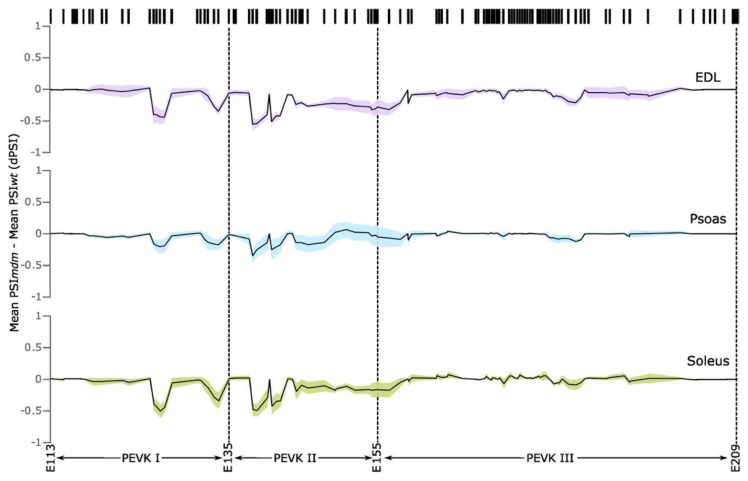
∆PSI plots for EDL (purple), psoas (blue), and soleus (green) highlighting that the major splice variation occurs in the PEVK region. The PSI values of the *mdm* titin exons were subtracted from the WT PSI values to determine the PSI difference (dPSI). Negative PSI values indicate a higher rate of exon inclusion in WT titin.

Most splicing changes were observed in exons encoding the intrinsically disordered PEVK region of *mdm* titin. PEVK is the most splicing-dense region of titin [5] and is therefore the most variable region among the isoforms. Nagy et al. classified this protein region into three segments that are each ~700 residues in length (GenBank accession: X90569.1), with the regions exhibiting an increase in persistence length from the N-terminus to C-terminus [45]. The orthologous regions of the mouse PEVK nucleotide sequence (GenBank accession: BN001114.1) were identified using protein-to-nucleotide alignments generated through tblastn. PEVKI was identified as exons 112–135, PEVKII as exons 135–155, and PEVKIII as exons 155–209. The PSI indices were calculated for each exon in these three regions to determine whether *mdm* muscles show a change in splicing compared to WT muscles. PSI indices showed significant differences between regions and more fluctuation in PEVKI (significant downregulation of exons 124–127 and 133–134 compared to WT; two-way ANOVA, all *p* < 0.0001) and PEVKII (significant downregulation of exons 138–145 in *mdm* compared to WT; two-way ANOVA, all *p* < 0.0029) than PEVKIII (significant downregulation of exon 194 in *mdm* compared to WT; two-way ANOVA, *p* = 0.0002) when the values between *mdm* and WT muscles were compared (Figure 2). The average PSI indices in these regions were lower in *mdm* compared to WT muscles (Figure 3). The most significant downregulation (comparatively lower PSI indexes) was observed in PEVKI and PEVKII, which appear to be splicing hotspots in *mdm* muscles.

Among the three muscles, the psoas showed the fewest splicing changes in PEVK, whereas *mdm* EDL and soleus showed the most splicing alterations relative to WT muscles. PEVK exons that showed significant differences in expression among muscles included exons 126, 127, 134, 139, 144, and 145 (two-way ANOVA, all *p* < 0.02). Splicing alterations in the PEVK region started to accumulate at exon 124 (Figure 2). EDL showed the largest reduction in exon inclusion among the three *mdm* muscles. PEVKI exons 124–127 (coding for PEVK14-17 domains) and exons 133–134 (coding for PEVK23-24 domains) exhibited a drop in PSI indexes in *mdm* EDL and soleus compared to WT muscles. The *mdm* EDL showed lower splicing indexes compared to WT throughout the PEVKII range, which continued into the PEVKIII region up to exon 159 (PEVK49 domain). Changes in the PSI indices in PEVKII of *mdm* soleus muscle started at the same exon as EDL, but the expression drop was condensed into two regions: exons 138–143 (PEVK28-33 domains) and exons 150–153 (PEVK40-43 domains). The *mdm* psoas did not show large changes in splicing in PEVKI, but PEVKII exons 138 and 139 (PEVK28 and 29 domains) showed a downregulation of expression. In general, PEVKIII exon expression changed the least with *mdm* deletion. The exons coding for the middle part of PEVKIII had <0.25 PSI values in both WT and *mdm* muscles in the EDL, psoas, and soleus, except for exon 172, which encodes the PEVK75 domain. This exon exhibits an expression spike with a ~0.75 PSI value in all three muscles, with no expression difference between *mdm* and WT muscles. This exon encodes a PPAK-rich domain in PEVKIII. Expression downregulation in the PEVKIII region was observed in exons 188–195 (PEVK93-100 domains) in EDL, but this was restricted to exons 194–195 (PEVK99-100 domains) in the psoas, and no clear expression change was observed in soleus PEVKIII.

The change in mass of the PEVK region was estimated using the observed PSI values for each exon. Exons with PSI = 1 were included in all calculations and exons with PSI = 0 were excluded. We then calculated the potential mass three ways to account for PSI values that were not 0 or 1. First, we included all exons that had PSI > 0 to calculate the maximum possible mass of the PEVK region. Second, we excluded all exons that had a PSI < 1 to determine the minimum possible mass of the PEVK region. Finally, to determine an intermediate mass, we multiplied the mass of each exon by the PSI value to scale the mass based on the percentage of inclusion of each exon. The intermediate mass was thought to be the most reliable estimate of the PEVK mass since it is likely that not all exons will be included or excluded in any particular transcript. Using these values, the PEVK mass of WT EDL was estimated to be 161 kDa, whereas that of *mdm* EDL was estimated to be 128 kDa, or 33 kDa smaller. For WT psoas, the PEVK mass was predicted to be 191 kDa and that of *mdm* psoas was 176 kDa, with a difference of 15 kDa. Finally, the WT soleus was predicted to be 207 kDa, whereas the *mdm* soleus was predicted to be 172 kDa, with a difference of 35 kDa. Overall, the lower PSI indices in *mdm* muscles compared to WT suggest that *mdm* muscles express shorter PEVK regions, which, although too small to be visualized using gel electrophoresis, are predicted to lead to the expression of shorter and stiffer titin isoforms in *mdm* muscles compared to WT.

#### 2.1.3. Titin C-Terminus Splicing

**Figure 4:**
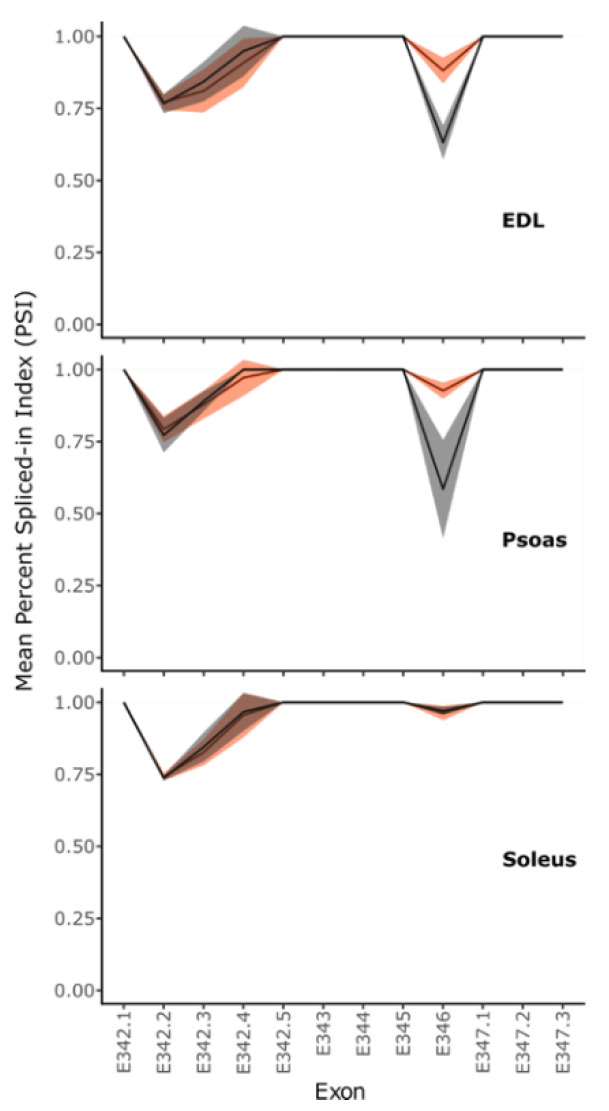
Expression of MEx5 (exon 346) is upregulated in fast *mdm* muscles (two-way ANOVA, *p* < 0.0001). Mean PSI values calculated for MEx1-6 encoding exons are shown. PSI = 0—complete exclusion and PSI = 1–100% inclusion of the exon/exon segment into the expressed transcript pool. The shaded area shows the standard deviation of mean PSI values. Exon labels are suffixed with a period followed by a number if the region was split into separate segments based on the variable distribution of reads in the alignments.

MEx domains play an important role in anchoring the titin C-termini from the opposite half sarcomeres at the M-line. The expression of exon 346, which codes for the MEx5 domain, was upregulated in *mdm* fast muscles (EDL and psoas) compared to WT muscles but not in the slow soleus muscle (two-way ANOVA, all *p* < 0.0001; Figure 4). WT psoas and EDL had a PSI index of ~0.6 for MEx5 (EDL = 0.63, psoas = 0.58), whereas WT soleus had a PSI index of 0.97. Although no change in expression was observed in *mdm* soleus, the PSI index of MEx5 in psoas and EDL titin increased to ~0.9 (EDL = 0.88, psoas = 0.93) in *mdm* muscles. An increase in the inclusion of C-terminal domains in titin may indicate increased tethering strength at the M-line, which correlates with the expression changes at the Z-line of *mdm* titin. If the expressed titin isoforms are shorter, the anchoring domains of the molecules may need extra support, which could be the reason N- and C-termini of titin show higher exon inclusion in *mdm* titin transcripts of fast muscles in contrast to the lower exclusion in WT muscles.

#### 2.1.4. Extracellular Matrix/Collagen

**Figure 5:**
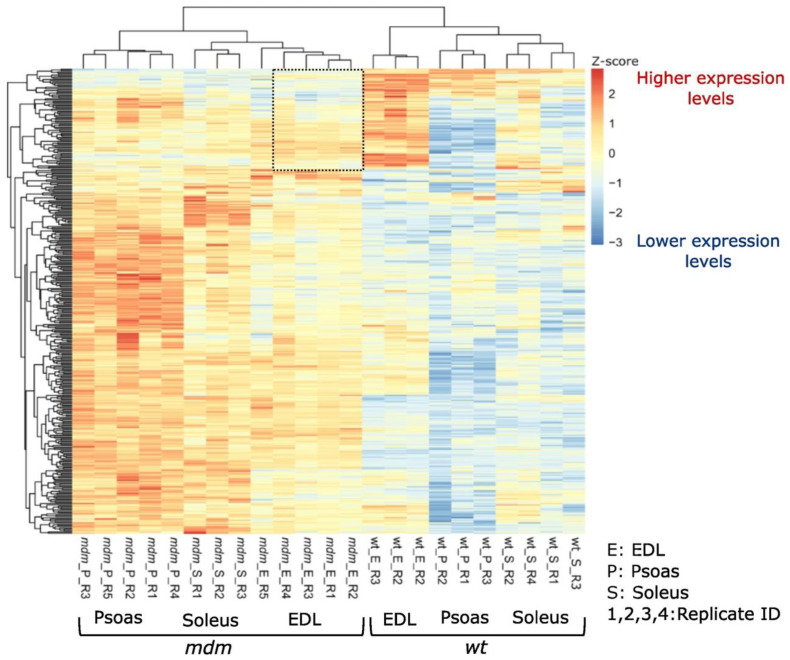
Gene expression changes in ECM-associated genes in *mdm* skeletal muscles compared to WT muscles. The expression heatmap was generated using ECM-associated differentially expressed genes identified from the RNA sequencing data. The heatmap was generated using Z-scores calculated for each gene in each muscle using variance-stabilized transformed expression levels. Regions containing downregulated genes in *mdm* EDL muscles compared to WT EDL are highlighted with dotted lines.

Comparative gene expression analysis for *mdm* vs. WT muscles showed significant expression changes in genes associated with a wide array of cellular functions [46]. Among these changes, expression shifts in extracellular matrix (ECM) genes were investigated as they are closely associated with muscle passive tension. *Mdm* muscles exhibited unique expression signatures, with ECM organization and collagen biosynthesis transcripts downregulated in *mdm* EDL, whereas ECM-related transcripts were upregulated in the *mdm* psoas and soleus (Figure 5). These observations are especially interesting as two fast muscles (EDL and psoas) have been reported to exhibit different changes in muscle passive tension in response to the *mdm* deletion [19]. Taken together, these results are broadly consistent with the high passive tension observed in different *mdm* muscles.

### 2.2. Mechanical Experiments

**Figure 6:**
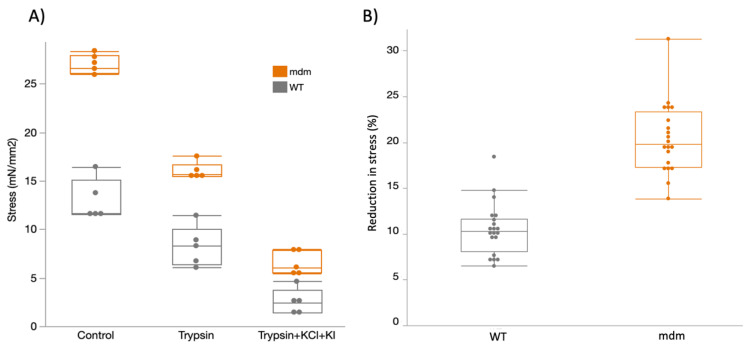
(**A**) Passive tension of *mdm* fiber bundles was significantly higher than WT for untreated control (ANOVA; F = 131.6; *p* < 0.0001), trypsin-treated (ANOVA; F = 45.5; *p* < 0.0001), and trypsin + KCl + KI-treated (ANOVA; F = 22.7; *p* = 0.0014) fiber bundles. Passive tension decreased significantly from control to trypsin-treated fiber bundles in both WT and *mdm* (ANOVA; F = 419.7; *p* < 0.0001). In addition, there was a significant decrease in passive tension after trypsin + KCl + KI in both WT (ANOVA; F = 142.82; *p* < 0.0001) and *mdm* fiber bundles (ANOVA; F = 29.45; *p* = 0.0006). All six groups differed significantly from each other (Tukey’s HSD, *p* < 0.05). (**B**) Reduction (%) in passive stress after trypsin + KCl + KI treatment was significantly greater in *mdm* soleus fiber bundles compared to WT (ANOVA; F = 64.1; *p* < 0.0001), suggesting a higher titin-based passive tension in *mdm* muscle.

Before treatment (control), the steady-state stress (mN/mm^2^) after passive stretch from 2.6 to 3.0 μm (Figure 6) was greater in *mdm* (n, mean ± s.d.; 20, 27.0 ± 0.97 mN/mm^2^) than in WT soleus fiber bundles (21, 13.0 ± 2.14 mN/mm^2^; ANOVA; F = 131.6, *p* < 0.0001). After trypsin treatment, passive tension remained higher in *mdm* (16.1 ± 0.89 mN/mm^2^) compared to WT fiber bundles (8.3 ± 2.1 mN/mm2; ANOVA; F = 45.5; *p* < 0.0001). After treatment with trypsin + KCl + KI (Figure 6A), *mdm* (6.7 ± 1.27 mN/mm^2^) fiber bundles also had significantly higher passive tension than WT (2.6 ± 1.3 mN/mm^2^; ANOVA; F = 22.8; *p* = 0.0014). The passive tension remaining after treatment with trypsin + KCl + KI represents the collagen-based passive tension. Passive stress decreased significantly from treatment with trypsin to treatment with trypsin + KCl + KI (Figure 6A) in both *mdm* (ANOVA; F = 142.82; *p* < 0.0001) and WT fiber bundles (ANOVA; F = 29.45; *p* = 0.0006). *Mdm* fiber bundles (20.4 ± 1.24 mN/mm^2^) had a significantly larger reduction in passive stress after trypsin + KCl + KI treatment (titin-based passive stress) than WT (Figure 6B; 10.5 ± 1.53 mN/mm^2^; ANOVA; F = 64.1; *p* < 0.0001).

## 3. Discussion

Two primary factors modulate passive stiffness in skeletal muscles; structural and functional properties of titin and extracellular matrix (ECM) components, especially collagen content [32]. The contribution of these two factors to total passive stiffness varies among skeletal muscles [15]. Previous studies demonstrated higher passive tension in single fibers [21], fiber bundles [20], and whole muscles from *mdm* mice [23,25]. Based on the increased passive tension and lower extensibility of *mdm* muscles, it has been suggested that the *mdm* deletion may also lead to the expression of shorter splice variants through exon skipping events [30] or the indirect reduction by post-translational processing of PEVK domains [20]. Here we present evidence of titin splicing as a crucial contributor to the increased passive tension of *mdm* muscles and muscle fibers and the first attempt to study alternative splicing changes in *mdm* titin. These data bridge a critical gap in the information necessary for understanding titin structure and function in *mdm* muscles [19].

The results from RNA sequencing identified key splicing changes in titin that are concentrated in Z-repeat 5-6, PEVK, and Mex5 exons in *mdm* muscles. Exons located at the N- and C-terminal domains (Z-line and M-line) tended to have higher inclusion rates in *mdm* titin transcripts, whereas PEVK exons showed an overall decrease in expression compared to WT. As proximal I-band and PEVK exons are the primary determinants of titin isoform size [5], the lower PSI values in these domains correspond to the expression of shorter splice variants in *mdm* muscles. The expression of shorter titin isoforms aligns with the increased passive tension and lower passive extensibility observed in *mdm* muscles (see below).

Sarcomere Z-band thickness is linked to the fiber type of skeletal muscles [47]. Fast muscles express thinner Z-bands and slow muscles express wider Z-bands by adjusting the expression of Z-repeats 4-6 [48]. The thickness of the Z-band is determined by the number of interaction points between titin and thin filaments, which is secured through α-actinin [48]. Each repeat has a variable affinity to α-actinin, with Z-repeats 4, 5, and 6 having the weakest affinities [49,50]. The expression ratio between relatively strong and weak Z-repeats affects titin’s anchoring strength in the sarcomere and modulates a muscle’s ability to withstand and transmit tension between adjacent sarcomeres [22,47]. Every other Z-repeat in titin interacts with α-actinin [49]. By splicing out low-affinity Z-repeats, fast muscles can withstand the higher forces produced by stiffer titin isoforms. In contrast, the expression of longer Z-repeats in slow muscles weakens the overall anchoring strength. However, longer Z bands in titin may also increase the number of transient bonds despite the inclusion of weaker regions.

As titin proteins are tethered between neighboring sarcomeres through α-actinin, we also investigated the expression of α-actinin 1–4 genes (Figure 1B). α-actinin-1 and 4 are “non-muscle” isoforms that engage in actin cross-linking in the cytoskeleton [51,52]. α-actinin-1 is associated with cell membranes at the ends of actin stress fibers and adherent junctions [53]. Isoform 4 is found in the cytoplasm and the nucleus [53]. α-actinin-2 and 3 are muscle isoforms crucial for actin and titin cross-linking at the sarcomere Z- disc [45,53]. α-actinin-2 is found in all skeletal muscles but isoform 3 is predominantly expressed in fast muscles [53]. The increased expression of muscle-specific α-actinin-2 agrees well with the increased expression of more Z-repeats at the N-terminus of fast muscle titin, which requires more α-actinins to secure the assembly of titin to actin. The reduced expression of α-actinin-3 in *mdm* psoas may indicate fiber-type switching, as α-actinin-3 is predominantly expressed in fast muscles [35]. The deficiency of α-actinin-3 is linked to weaker muscle strength and power generation, thus increasing susceptibility to eccentric damage [54]. Furthermore, α-actinin-3 knockout mice have shown significant decreases in grip strength, muscle mass, and type 2B fiber size, and an increase in muscle aerobic capacity [54]. Therefore, it has been suggested that alterations in α-actinin levels can significantly alter Z-disc protein dynamics, affecting sarcomere integrity [54]. Similarly, the Mex5 exon is characteristically expressed in healthy slow muscles [55,56], but *mdm* fast muscles show a significant increase in Mex5 in titin transcripts. Overall, these differences suggest fiber-type switching in fast muscles. Lopez et al. [20] found that the expression of fast MHC isoforms decreased from 2 to 6 weeks of age in the *mdm* diaphragm, supporting the hypothesis that the *mdm* mutation induces a shift toward slower isoforms. However, additional evidence (e.g., from immunohistochemistry) is needed to definitlvely test the hypothesis that *mdm* muscles shift toward slower fiber types.

The PEVK domain of titin showed high levels of exon exclusion (lower PSI) in *mdm* muscles. PEVK accounts for most of the titin-based passive tension in skeletal muscles [14,19,57]. Different segments of PEVK have differential extensibility, which decreases from the N- to C-terminus [45]. Moreover, PEVK segments have different local affinities for F-actin [39] based on the poly-E motif composition of the segment [52]. N-terminal PEVK segments carry a higher percentage of poly-E motifs and thus contribute more to viscous drag in unloaded muscle shortening [45]. Glutamate residues in poly-E motifs also play a critical role in the Ca2+/S100A1-dependent loss of bending rigidity of the PEVK regions in activated muscles [58]. Therefore, the composition of PEVK and which exons are included in specific isoforms appear to contribute to skeletal muscle passive tension [14].

The expression of shorter isoforms increases the passive stress and decreases the extensibility of titin [20], but the loss of poly-E-containing PEVK motifs could increase titin’s compliance. Competition between these factors along with the collagen content determine the overall passive tension of *mdm* muscles. Although the passive tension increase in *mdm* muscles was previously attributed solely to increased collagen content [19,21], the current study shows that the increase in exon splicing and the changes in collagen content have collective but variable contributions in different *mdm* muscles. It is hard to quantify the relative contributions of PEVK splicing and increasing collagen content to passive tension from the gene expression data alone because *mdm* muscles differed in the expression of both PEVK splicing and extracellular matrix-associated genes.

We found that the degree of PEVK exon exclusion decreased from SOL > EDL > psoas. *Mdm* psoas had the fewest splicing changes in the PEVK region compared to *mdm* EDL and SOL, which had significantly greater exon exclusion in the PEVK region, particularly for exons 126, 127, 134, 139, and 145 (two-way ANOVA, all *p* < 0.0029). It remains unclear why the SOL and EDL exhibit similar splicing changes in PEVK in contrast to the psoas, which was much less affected. Because a shorter PEVK region will result in higher passive tension [15], these results are consistent with the patterns of variation in *mdm* passive tension observed in previous studies. For example, Powers et al. found no difference in stiffness between passively stretched *mdm* and WT psoas myofibrils [22], which is consistent with the predicted small (~15 kDa) difference in WT versus *mdm* PEVK exon splicing observed here. Both Hessel et al. [23] and Tahir et al. [23] found higher passive stress in *mdm* compared to WT soleus fiber bundles and intact muscles, respectively. Hessel et al. found higher passive tension in intact *mdm* EDL muscles compared to *mdm* soleus muscles [24], which is consistent with the relative amounts of exon exclusion in the PEVK region of titin. The PEVK region in *mdm* EDL titin is predicted to be ~128 kDa, whereas the soleus PEVK region in *mdm* titin is predicted to be ~172 kDa. This difference in exon inclusion would account for the higher passive tension of intact *mdm* EDL muscles measured by Hessel et al. [24].

The second part of this study focused on investigating the relative contributions of titin and collagen to the passive tension of *mdm* soleus muscle fiber bundles. We found that (1) untreated *mdm* fiber bundles had significantly higher passive stress than WT fibers; (2) the reduction in passive stress after trypsin + KCl + KI treatment (titin-based stress) was significantly greater in *mdm* fiber bundles compared to WT fibers; and (3) the stress remaining after trypsin + KCl + KI treatment (collagen-based stress) was also higher in *mdm* than in WT fiber bundles. Consistent with the results from RNAseq, these results demonstrate that the higher total passive tension in *mdm* compared to WT is due to the contributions of both titin and collagen.

Before treatment with trypsin + KCl + KI, steady-state tension after passive stretch (see Figure 6) was significantly higher in control (untreated) *mdm* soleus fiber bundles compared to WT fiber bundles from soleus muscles. Earlier studies also found higher passive stress in *mdm* fibers [31] and intact muscles [25] compared to WT. This finding is consistent with previous studies, which also report higher passive stress in *mdm* muscles compared to WT [21,25,59]. The higher total passive stress could be due to higher titin passive tension resulting from alterations in the exon splicing in the PEVK region of titin that could lead to the expression of shorter and stiffer titin isoforms. A previous study also found that the deletion of a small number of exons in the proximal IG region of titin led to alternative splicing of PEVK exons in a transgenic mouse model [17]. In contrast, the deletion of 46 exons (75% of the contour length) in the PEVK region produced no apparent change in exon splicing [10].

Our results demonstrate that the reduction in passive stress after treatment with trypsin + KCL + KI (titin-based passive stress) was larger in *mdm* soleus fiber bundles compared to WT (see Figure 6). The finding that *mdm* soleus fiber bundles have a larger reduction in passive stress compared to WT after all the treatments supports the results of the transcriptomic analysis that demonstrated splicing of titin exons in *mdm* muscles. We predict that altered splicing patterns in *mdm* soleus likely lead to a shorter I-band region of titin and contribute to the higher passive tension of *mdm* fibers compared to WT.

We also found that greater passive tension remained in *mdm* soleus fiber bundles than in WT after the degradation of titin by trypsin + KCL/KI. This remaining passive tension in the fiber bundles is the collagen-based tension (see Figure 6). This result is consistent with a previous study by Powers et al. (2017), which found higher collagen content in *mdm* psoas fibers compared to WT, demonstrated via a colorimetric hydroxyproline assay [21]. Future studies should aim toward quantifying and comparing the protein (e.g., collagen, a-actinin, MHC, etc.) content of the different *mdm* muscles studied here using Western blot or similar techniques. The results of the current study support titin as a significant contributor to higher passive tension in *mdm* muscles, in addition to collagen [20,60].

## 4. Materials and Methods

A B6C3Fe a/a-Ttn*mdm*/J mouse colony was established at the animal care facility of Northern Arizona University, Flagstaff, AZ, USA, using mice obtained from The Jackson Laboratory (Bar Harbor, ME, USA). Mice were fed ad libitum and maintained in a temperature-controlled facility in a light: dark 12hr:12hr cycle. Mice were sacrificed via isoflurane overdose confirmed by cervical dislocation. Protocols for use of animals were approved by the Institutional Animal Care and Use Committee at NAU (Reference number:18-002).

### 4.1. RNA Sequencing

*Sample preparation:* Samples were extracted from 40 ± 6-day-old mice (n = 23). Three wild-type EDL, wild-type psoas, and *mdm* soleus; four wild-type soleus; and five *mdm* EDL and *mdm* psoas muscles were stored in an RNAlater™ stabilization solution (ThermoFisher Scientific, Waltham, MA, USA) at −80 °C. Frozen muscle samples were thawed, and total RNA was extracted using a Qiagen fibrous tissue total RNA extraction mini kit following the recommended protocol with DNAse treatment. When RNA was extracted without on-column DNAse treatment, a separate RNA treatment was carried out on the RNA samples using Promega RNase-Free DNase1. The concentration of extracted RNA was measured using a Qubit RNA Broad-Range assay, and RNA integrity numbers (RIN) were determined using an Agilent 2100 Bioanalyzer RNA 6000 Nano assay. Samples with concentrations >20 ng/μL and RIN > 7 were subsequently used to prepare cDNA libraries using an Illumina TruSeq Stranded Total RNA Library Prep Kit. Library quality was determined using KAPA Library Quantification qPCR Kit for Illumina sequencing platforms and library sizes were determined using an Agilent Bioanalyzer 2100 High Sensitivity dsDNA quantification assay. The mean average library sizes of the samples were 256–323 bp (Tables S1 and S2). The cDNA libraries were sequenced using an Illumina NextSeq 500 High Throughput sequencer, and 75 bp paired-end reads were generated over five sequencing runs. The sequenced library coverage varies between 9.5 and 100 million reads with a median of 23 million (Tables S1 and S2).

*Raw data processingand alignment:* Sequencing data quality was assessed using the FastQC v0.73 quality control tool for high-throughput sequence data (http://www.bioinformatics.babraham.ac.uk/projects/fastqc/, accessed on 5 January 2020). Libraries showing a quality score <20 at any given position along the read length were further processed using a sliding window quality filter (window size 4) to remove low-quality bases using Trimmomatic v0.32 [61]. Following this step, the paired-end reads collected from the preprocessing step were used for the subsequent data analysis (Tables S1 and S2). Samples with a quality score >20 were used for the subsequent analysis. Adapter sequences used for sequencing were removed while converting initial BCL data to fastqc from the sequencing center, and no adapter contamination was detected in FASTQC analysis.

Preprocessed data were aligned to the *Mus musculus* GRCm38.p4 genome using the following steps. Insert sizes between paired-end reads were calculated using a partial read alignment step carried out using the Galaxy online platform [57]. A subset of 250,000 reads from each sample was aligned to the mm10 reference mouse genome available in the Galaxy web platform, keeping the parameters at the default settings. Alignment statistics were generated using the CollectInsertSizeMetrics Picard tool v2.18.2.1 (http://broadinstitute.github.io/picard/, accessed on 2 May 2020). Calculated average insert sizes and standard deviations were then used to generate complete read alignments using Tophat v2.1.1, keeping the other parameters at the default values [62] (see alignment details in Tables S1 and S2).

*Splicing index calculation and data visualization:* Generated read alignments were used as input to calculate the percent spliced-in indexes for titin exons. Reference genome data for the titin gene was extracted from *Mus musculus* GRCm38.100 to improve exon annotations. Calculations were carried out following Schafer et al. [63]. Calculated PSI indexes were assigned to “counting bins,” which were defined while the reformatting step was carried out at the beginning of the published protocol. Therefore, the exon numbers were manually assigned to counting bins using the annotated exon boundaries in ensemble titin isoform ENSMUST00000099981.9 and domain sequence annotations in *Mus musculus* titin GenBank entry BN001114.1. Sequence alignments were conducted using the blastn sequence alignment tool with default parameters.

Graphical outputs were generated using R libraries ggplot2, regioneR, GenomicRanges, and karyoploteR. Average PSI indexes and standard deviations for each *mdm* and wild-type muscle were calculated using R library matrixStats. Mean PSI index differences (dPSI) were calculated by subtracting the mean WT PSI index from the mean *mdm* PSI index for each exon, separately for each muscle. Standard deviations were calculated using the equation SD = sqrt{[(n_wild-type_ − 1) * (s_wild-type_^2^) + (n_mdm_ − 1) * (s_mdm_^2^)]/(n_wild-type_ + n_mdm_ − 2)} for small sample sizes (<30) assuming equal population standard deviations (n = number of samples, s = standard deviation).

*Polymerase chain reaction and agarose gel electrophoresis:* Approximately 200ng of starting RNA was used to generate the cDNA first strand using Protoscript II reverse transcriptase and Invitrogen random primers (catalog number 48190011) following the recommended temperatures and times for reverse transcription reaction [153 pmol/µL Random Primer 1 µL, 10 mM dNTP 1 µL, 5X ProtoScript II Buffer 4 µL, 0.1 M DTT 2 µL, ProtoScript II-RT, 1 µL, and Nuclease-free H_2_O]. PCR reactions were carried out to amplify exons 11–13 using flanking primers on exon10 and 14 (F:TCTCCGCAACCAAAGCCAAA, R:CTCCACATGCGTAGGCTCTC) using 1 μL cDNA. The protocol recommended by Invitrogen Platinum SuperFi DNA Polymerase (catalog number 12351010) was used to carry out PCR reactions (initial denaturation at 98 °C for 45 s; 35 amplification cycles—denaturation at 98 °C for 30 s; primer annealing at 55 °C; extension at 72 °C for 1min; and final extension at 72 °C for 5 min). Amplified products were separated on a 1% agarose gel and stained using SYBR Safe DNA Gel Stain (catalog number S33102).

*Data analysis:* Statistical analysis was performed using JMP Pro15 software. Two-way factorial analysis of variance (ANOVA) was used to analyze the effects of genotype (WT, *mdm*), muscle (EDL, psoas, soleus), and the genotype x muscle interaction on the percent spliced in (PSI) for a total of 142 transcripts in five regions of titin (Z-repeats = exons 10–14, PEVK1 = exons 113–135, PEVK2 = exons 135–155, PEVK3 = exons 156–209, and MEx5 = exons 342 and346). Exons 344, 345, and 347 showed no variability among genotypes or muscles (PSI = 1) and were excluded from the analysis. Due to the large number of statistical tests (n = 142 transcripts), the sequential Bonferroni test was used to reduce the likelihood of Type 2 errors (rejecting the null hypothesis when it is true).

### 4.2. Mechanical Testing

WT mice (n = 5) at 30–36 days of age and *mdm* mice (n = 5) at 15–20 days of age were used for the mechanical experiments. We used fiber bundles from younger mice to reduce the probability of fibrosis [20] and degeneration [60], which might affect the active and passive tension after stretch. SOL muscles were extracted from euthanized mice following standard procedures [64].

*Preparation of skinned fiber bundles:* Skinned soleus fiber bundles (n = 4–5 bundles per muscle) were prepared using standard techniques to minimize the contributions of endomysium to passive tension [65]. Extracted muscles were placed in a collection solution (50 mM Tris, 100 mM KCL, 2 mM MgCl_2_, 1 mM EGTA, pH 7.0) for 6–7 h at 4 °C, then transferred to an overnight solution (50 mM Tris, 2 mM KCl, 100 mM NaCl, 2 mM MgCl_2_, 1 mM EGTA, pH 7.0) with 1:1 glycerol for 12 h at 4 °C, and finally placed in long-term storage solution (collection solution with 1:1 glycerol) for 4–6 weeks at −20 °C [66]. To protect against protein degradation, protease inhibitor (PI) tablets (Complete^®^, Roche Diagnostics, Montreal, QC, Canada) were added to all solutions (1 tablet per 50 mL solution). Muscles were used 4–6 weeks after surgery. On the day of the experiments, muscles were washed in a relaxing solution (170 mM potassium propionate, 2.5 mM magnesium acetate, 20 mM MOPS, 5 mM K_2_EGTA, and 2. 5 mM ATP, pH 7.0) to remove the rigor-glycerol solution. Bundles of 4–5 fibers were dissected from each muscle in a petri dish filled with ice-cold relaxing solution and used immediately for experimentation. The solutions used during the experiments included relaxing solution containing 0.6 M KCl (no PI), relaxing solution containing 1.0 M KI (no PI), and relaxing solution with 0.25 µg/mL trypsin (no PI), all at pH 7.0 [32]. All experiments were conducted at room temperature (22 °C).

Using acetone and cellulose acetate (Merck, Darmstadt, Germany), each fiber bundle was glued lengthwise to a high-speed length controller on one end (Aurora Scientific Inc., model 322C, Aurora, ON, Canada), and a force transducer on the other end (Aurora Scientific Inc., model 400A, Aurora, ON, Canada). Length control and force measurements were accomplished using an ASI802D data acquisition and control system (Aurora Scientific Inc., Aurora, ON, Canada). The rig was connected to an inverted microscope (Leica, DMIRE2) with an ocular micrometer for the measurement of the fiber diameter (graticule 10 mm = 100 divisions). The microscope was fitted with a sarcomere length (SL) tracking camera (Aurora HVSL600A). Changes in SL were measured via SL tracking and acquisition software. The fiber rig was controlled using Linux software, which switches wells containing different solutions. For measurement of sarcomere length, the Aurora 901B camera has a spatial resolution of 5 pixels/µm at high magnification (50× optical lens of the inverted microscope) and 2 pixels/µm at low magnification (20× optical lens of the inverted microscope) at 300 frames per second full frame (Aurora Scientific Inc., Aurora, ON, Canada, 2012).

A pre-conditioning stretch in relaxing solution was performed before starting each experiment to prevent slippage or breakage of fibers. Passive tension was measured by passively stretching the fiber bundles from 2.6 to 3.0 µm at 0.04 µm/s and then holding them isometrically for 60 s until the steady-state force was attained. Passive tension was measured under four conditions: (1) in relaxing solution only; (2) after treatment with trypsin for 35 min to degrade titin; (3) after treatment with KCl for 45 min to remove the thick filament anchors; and (4) after treatment with KI for 45 min to remove the thin filament anchors. After each treatment, the fiber bundles were rested for 7 min and then washed for 2 min in relaxing solution with PI to stop further protein degradation before the next treatment began.

Stress (mN/mm^2^) was calculated by dividing the force by the cross-sectional area, assuming a cylindrical shape of the fiber bundles. After the extraction protocol, the fiber bundles were re-stretched at 0.04 µm/s. The passive force remaining after trypsin + KCl + KI treatment was assumed to be collagen-based. The absolute reduction in passive stress after all treatments (titin-based passive force) was calculated as the difference between the total passive force before vs. after treatment with trypsin + KCl + KI [17,32].

*Data analysis:* Statistical analysis was performed using JMP Pro14 software. The Shapiro–Wilk test showed that the data were normally distributed (*p* > 0.05). Levene’s test showed that the variances differed significantly between genotypes (*p* < 0.05). We corrected the data with non-homogenous variances using Box–Cox transformation. One-way ANOVA with genotype as the main effect was used to evaluate the differences between the WT (n = 5 muscles and 21 total fibers) and *mdm* fiber bundles (n = 5 muscles and 20 fiber bundles). Because more than one fiber bundle from a single muscle was included in the analysis, we accounted for the within-muscle variation by including a random effect of muscle nested within the genotype. One-way ANOVA showed that the random effect of variation among muscles was significant for all dependent variables in both genotypes (WT and *mdm*). Alpha values were set at 0.05. The dependent variables were control passive stress at 3.0 µm, passive stress at 3.0 µm after trypsin treatment, passive stress after KCL/KI treatment (collagen-based force), and titin-based passive forces in fiber bundles from 2.6 to 3.0 µm. Data are presented as mean ± standard deviation (s.d.).

## Data Availability

All raw data is available upon request.

## Supplementary Materials

The supporting information can be downloaded at: https://www.mdpi.com/article/10.3390/ijms23168858/s1.

## References

[1] Savarese M., Sarparanta J., Vihola A., Udd B., Hackman P. (2016). Increasing Role of Titin Mutations in Neuromuscular Disorders. J. Neuromuscul. Dis..

[2] Maruyama K. (1976). Connectin, an elastic protein from myofibrils. J. Biochem..

[3] Wang K., McClure J., Tu A. (1979). Titin: Major myofibrillar components of striated muscle. Proc. Natl. Acad. Sci. USA.

[4] Labeit S., Barlow D.P., Gautel M., Gibson T., Holt J., Hsieh C.L., Francke U., Leonard K., Wardale J., Whiting A. (1990). A regular pattern of two types of 100-residue motif in the sequence of titin. Nature.

[5] Guo W., Bharmal S.J., Esbona K., Greaser M.L. (2010). Titin diversity—Alternative splicing gone wild. J. Biomed. Biotechnol..

[6] Bang M.L., Centner T., Fornoff F., Geach A.J., Gotthardt M., McNabb M., Witt C.C., Labeit D., Gregorio C.C., Granzier H. (2001). The complete gene sequence of titin, expression of an unusual approximately 700-kDa titin isoform, and its interaction with obscurin identify a novel Z-line to I-band linking system. Circ. Res..

[7] Gautel M., Goulding D. (1996). A molecular map of titin/connectin elasticity reveals two different mechanisms acting in series. Febs Lett..

[8] Linke W.A., Ivemeyer M., Mundel P., Stockmeier M.R., Kolmerer B. (1998). Nature of PEVK-titin elasticity in skeletal muscle. Proc. Natl. Acad. Sci. USA.

[9] Bartoo M.L., Linke W.A., Pollack G.H. (1997). Basis of passive tension and stiffness in isolated rabbit myofibrils. Am. J. Physiol..

[10] Brynnel A., Hernandez Y., Kiss B., Lindqvist J., Adler M., Kolb J., Van der Pijl R., Gohlke J., Strom J., Smith J. (2018). Downsizing the molecular spring of the giant protein titin reveals that skeletal muscle titin determines passive stiffness and drives longitudinal hypertrophy. Elife.

[11] Granzier H.L., Labeit S. (2004). The giant protein titin: A major player in myocardial mechanics, signaling, and disease. Circ. Res..

[12] Wang K., McCarter R., Wright J., Beverly J., Ramirez-Mitchell R. (1993). Viscoelasticity of the sarcomere matrix of skeletal muscles. The titin-myosin composite filament is a dual-stage molecular spring. Biophys. J..

[13] Freiburg A., Trombitas K., Hell W., Cazorla O., Fougerousse F., Centner T., Kolmerer B., Witt C., Beckmann J.S., Gregorio C.C. (2000). Series of exon-skipping events in the elastic spring region of titin as the structural basis for myofibrillar elastic diversity. Circ. Res..

[14] Linke W.A., Ivemeyer M., Olivieri N., Kolmerer B., Rüegg C.J., Labeit S. (1996). Towards a molecular understanding of the elasticity of titin. J. Mol. Biol..

[15] Prado L.G., Makarenko I., Andresen C., Kruger M., Opitz C.A., Linke W.A. (2005). Isoform diversity of giant proteins in relation to passive and active contractile properties of rabbit skeletal muscles. J. Gen. Physiol..

[16] Neagoe C., Opitz C.A., Makarenko I., Linke W.A. (2003). Gigantic variety: Expression patterns of titin isoforms in striated muscles and consequences for myofibrillar passive stiffness. J. Muscle Res. Cell Motil..

[17] Buck D., Smith J.E., Chung C.S., Ono Y., Sorimachi H., Labeit S., Granzier H.L. (2014). Removal of immunoglobulin-like domains from titin’s spring segment alters titin splicing in mouse skeletal muscle and causes myopathy. J. Gen. Physiol..

[18] Garvey S.M., Rajan C., Lerner A.P., Frankel W.N., Cox G.A. (2002). The muscular dystrophy with myositis (mdm) mouse mutation disrupts a skeletal muscle-specific domain of titin. Genomics.

[19] Nishikawa K., Lindstedt S.L., Hessel A., Mishra D. (2020). N2A Titin: Signaling Hub and Mechanical Switch in Skeletal Muscle. Int. J. Mol. Sci..

[20] Lopez M.A., Pardo P.S., Cox G.A., Boriek A.M. (2008). Early mechanical dysfunction of the diaphragm in the muscular dystrophy with myositis (Ttnmdm) model. Am. J. Physiol. Cell Physiol..

[21] Powers K., Joumaa V., Jinha A., Moo E.K., Smith I.C., Nishikawa K., Herzog W. (2017). Titin force enhancement following active stretch of skinned skeletal muscle fibres. J. Exp. Biol..

[22] Powers K., Nishikawa K., Joumaa V., Herzog W. (2016). Decreased force enhancement in skeletal muscle sarcomeres with a deletion in titin. J. Exp. Biol..

[23] Hessel A.L., Joumaa V., Eck S., Herzog W., Nishikawa K. (2019). Optimal length, calcium sensitivity and twitch characteristics of skeletal muscles from mdm mice with a deletion in N2A titin. J. Exp. Biol..

[24] Hessel A.L., Nishikawa K.C. (2017). Effects of a titin mutation on negative work during stretch-shortening cycles in skeletal muscles. J. Exp. Biol..

[25] Tahir U., Monroy J.A., Rice N.A., Nishikawa K.C. (2020). Effects of a titin mutation on force enhancement and force depression in mouse soleus muscles. J. Exp. Biol..

[26] Huebsch K.A., Kudryashova E., Wooley C.M., Sher R.B., Seburn K.L., Spencer M.J., Cox G.A. (2005). Mdm muscular dystrophy: Interactions with calpain 3 and a novel functional role for titin’s N2A domain. Hum. Mol. Genet..

[27] Pace C.M., Mortimer S., Monroy J.A., Nishikawa K.C. (2017). The effects of a skeletal muscle titin mutation on walking in mice. J. Comp. Physiol. A Neuroethol. Sens. Neural. Behav. Physiol..

[28] Taylor-Burt K.R., Monroy J., Pace C., Lindstedt S., Nishikawa K.C. (2015). Shiver me titin! Elucidating titin’s role in shivering thermogenesis. J. Exp. Biol..

[29] Miyano C.A., Orezzoli S.F., Buck C.L., Nishikawa K.C. (2019). Severe thermoregulatory deficiencies in mice with a deletion in the titin gene TTN. J. Exp. Biol..

[30] Monroy J.A., Powers K.L., Pace C.M., Uyeno T., Nishikawa K.C. (2017). Effects of activation on the elastic properties of intact soleus muscles with a deletion in titin. J. Exp. Biol..

[31] Powers K., Schappacher-Tilp G., Jinha A., Leonard T., Nishikawa K., Herzog W. (2014). Titin force is enhanced in actively stretched skeletal muscle. J. Exp. Biol..

[32] Wu Y., Cazorla O., Labeit D., Labeit S., Granzier H. (2000). Changes in titin and collagen underlie diastolic stiffness diversity of cardiac muscle. J. Mol. Cell Cardiol..

[33] Labeit S., Kolmerer B. (1995). Titins: Giant proteins in charge of muscle ultrastructure and elasticity. Science.

[34] Luther P.K. (2009). The vertebrate muscle Z-disc: Sarcomere anchor for structure and signalling. J. Muscle Res. Cell Motil..

[35] Mills M., Yang N., Weinberger R., Vander Woude D.L., Beggs A.H., Easteal S., North K. (2001). Differential expression of the actin-binding proteins, alpha-actinin-2 and-3, in different species: Implications for the evolution of functional redundancy. Hum. Mol. Genet..

[36] Edlund M., Lotano M.A., Otey C.A. (2001). Dynamics of alpha-actinin in focal adhesions and stress fibers visualized with alpha-actinin-green fluorescent protein. Cell Motil. Cytoskelet..

[37] Fyrberg E., Kelly M., Ball E., Fyrberg C., Reedy M.C. (1990). Molecular genetics of Drosophila alpha-actinin: Mutant alleles disrupt Z disc integrity and muscle insertions. J. Cell Biol..

[38] Otey C.A., Carpen O. (2004). Alpha-actinin revisited: A fresh look at an old player. Cell Motil. Cytoskelet..

[39] Pavalko F.M., Burridge K. (1991). Disruption of the actin cytoskeleton after microinjection of proteolytic fragments of alpha-actinin. J. Cell Biol..

[40] Honda K., Yamada T., Endo R., Ino Y., Gotoh M., Tsuda H., Chiba H., Hirohashi S. (1998). Actinin-4, a novel actin-bundling protein associated with cell motility and cancer invasion. J. Cell Biol..

[41] Youssoufian H., McAfee M., Kwiatkowski D.J. (1990). Cloning and chromosomal localization of the human cytoskeletal alpha-actinin gene reveals linkage to the beta-spectrin gene. Am. J. Hum. Genet..

[42] Araki N., Hatae T., Yamada T., Hirohashi S. (2000). Actinin-4 is preferentially involved in circular ruffling and macropinocytosis in mouse macrophages: Analysis by fluorescence ratio imaging. J. Cell Sci..

[43] Nikolopoulos S.N., Spengler B.A., Kisselbach K., Evans A.E., Biedler J.L., Ross R.A. (2000). The human non-muscle alpha-actinin protein encoded by the ACTN4 gene suppresses tumorigenicity of human neuroblastoma cells. Oncogene.

[44] Hettige P., Tahir U., Nishikawa K.C., Gage M.J. (2020). Comparative analysis of the transcriptomes of EDL, psoas, and soleus muscles from mice. BMC Genom..

[45] Nagy A., Grama L., Huber T., Bianco P., Trombitas K., Granzier H.L., Kellermayer M.S.Z. (2005). Hierarchical extensibility in the PEVK domain of skeletal-muscle titin. Biophys. J..

[46] Hettige P., Tahir U., Nishikawa K., Gage M. (2022). Transcriptomic profile of muscular dystrophy with myositis in extensor digitorum longus, psoas, and soleus muscles from mice. BMC Genom..

[47] Sorimachi H., Freiburg A., Kolmerer B., Ishiura S., Stier G., Gregorio C.C., Labeit D., ALinke W., Suzuki K., Labeit S. (1997). Tissue-specific expression and alpha-actinin binding properties of the Z-disc titin: Implications for the nature of vertebrate Z-discs. J. Mol. Biol..

[48] Joseph C., Stier G., O’brien R., Politou A.S., Atkinson R.A., Bianco A., Ladbury J.E., Martin S.R., Pastore A. (2001). A structural characterization of the interactions between titin Z-repeats and the alpha-actinin C-terminal domain. Biochemistry.

[49] Grison M., Merkel U., Kostan J., Djinović-Carugo K., Rief M. (2017). α-Actinin/titin interaction: A dynamic and mechanically stable cluster of bonds in the muscle Z-disk. Proc. Natl. Acad. Sci. USA.

[50] Luther P.K., Squire J.M. (2002). Muscle Z-band ultrastructure: Titin Z-repeats and Z-band periodicities do not match. J. Mol. Biol..

[51] Kruger M., Linke W.A. (2009). Titin-based mechanical signalling in normal and failing myocardium. J. Mol. Cell Cardiol..

[52] Nagy A., Cacciafesta P., Grama L., Kengyel A., Málnási-Csizmadia A., Kellermayer M.S. (2004). Differential actin binding along the PEVK domain of skeletal muscle titin. J. Cell Sci..

[53] Oikonomou K.G., Zachou K., Dalekos G.N. (2011). Alpha-actinin: A multidisciplinary protein with important role in B-cell driven autoimmunity. Autoimmun. Rev..

[54] Seto J.T., Lek M., Quinlan K.G., Houweling P.J., Zheng X.F., Garton F., MacArthur D.G., Raftery J.M., Garvey S.M., Hauser M.A. (2011). Deficiency of alpha-actinin-3 is associated with increased susceptibility to contraction-induced damage and skeletal muscle remodeling. Hum. Mol. Genet..

[55] Chen Z., Maimaiti R., Zhu C., Cai H., Stern A., Mozdziak P., Ge Y., Ford S.P., Nathanielsz P.W., Guo W. (2018). Z-band and M-band titin splicing and regulation by RNA binding motif 20 in striated muscles. J. Cell Biochem..

[56] Kolmerer B., Olivieri N., Witt C.C., Herrmann B.G., Labeit S. (1996). Genomic organization of M line titin and its tissue-specific expression in two distinct isoforms. J. Mol. Biol..

[57] Afgan E., Baker D., Van den Beek M., Blankenberg D., Bouvier D., Čech M., Chilton J., Clements D., Coraor N., Eberhard C. (2016). The Galaxy platform for accessible, reproducible and collaborative biomedical analyses: 2016 update. Nucleic. Acids. Res..

[58] Labeit D., Watanabe K., Witt C., Fujita H., Wu Y., Lahmers S., Funck T., Labeit S., Granzier H. (2003). Calcium-dependent molecular spring elements in the giant protein titin. Proc. Natl. Acad. Sci. USA.

[59] Mishra D., Nishikawa K.C. (2022). Residual force enhancement is reduced in permeabilized fiber bundles from mdm muscles. J. Exp. Biol..

[60] Heimann P., Menke A., Rothkegel B., Jockusch H. (1996). Overshooting production of satellite cells in murine skeletal muscle affected by the mutation ″muscular dystrophy with myositis″ (mdm, Chr 2). Cell Tissue Res..

[61] Bolger A.M., Lohse M., Usadel B. (2014). Trimmomatic: A flexible trimmer for Illumina sequence data. Bioinformatics.

[62] Kim D., Pertea G., Trapnell C., Pimentel H., Kelley R., Salzberg S.L. (2013). TopHat2: Accurate alignment of transcriptomes in the presence of insertions, deletions and gene fusions. Genome. Biol..

[63] Schafer S., Miao K., Benson C.C., Heinig M., Cook S.A., Hubner N. (2015). Alternative Splicing Signatures in RNA-seq Data: Percent Spliced in (PSI). Curr. Protoc. Hum. Genet..

[64] Hakim C.H., Wasala N.B., Duan D. (2013). Evaluation of muscle function of the extensor digitorum longus muscle ex vivo and tibialis anterior muscle in situ in mice. J. Vis. Exp..

[65] Joumaa V., Herzog W. (2014). Calcium sensitivity of residual force enhancement in rabbit skinned fibers. Am. J. Physiol. Cell Physiol..

[66] Joumaa V., Rassier D.E., Leonard T.R., Herzog W. (2007). Passive force enhancement in single myofibrils. Pflug. Arch..

