# Supplementary material for: Contributions of Titin and Collagen to Passive Stress in Muscles from mdm Mice with a Small Deletion in Titin’s Molecular Spring
Source: Int J Mol Sci. 2022 Aug 9;23(16):8858. doi: 10.3390/ijms23168858 (PMC9408699; doi:10.3390/ijms23168858)
Supplement: Supplementary file 1 [file ijms-23-08858-s001.zip › ijms-1821135-supplementary.pdf]

Supplementary Material

Table S1. RNA-Seq read alignment summary for the wild-type samples used in the study.

		EDL_R1	EDL_R2	EDL_R3	Psoas_R1	Psoas_R2	Psoas_R3	Soleus_R1	Soleus_R2	Soleus_R3	Soleus_R4
Age (days)		42	54	44	42	54	44	42	44	32	44
RNA integrity number (RIN)		8.4	8.4	8.5	7.3	8.1	8	8.2	8.1	7.6	8.1
Library size		274	264	278	256	271	277	258	295	265	276
Left reads:	Initial number of reads	87185432	57368915	15186094	1E+08	45937372	13847980	22022374	28236087	12437526	15940351
	Minimum per base quality score (10th percentile)	14	14	27	14	14	32	32	32	32	32
	Percent filtered in (%)	81.38	80.83	N/A	81.86	80.26	N/A	N/A	N/A	N/A	N/A
	Alignment input	70950705	46369117	15186094	81953371	36870545	13847980	22022374	28236087	12437526	15940351
	Mapped	55572350	44539661	14774183	62419592	28077507	13534841	17953627	27374091	12109861	15593067
	Multiple alignments	18.5%	19.1%	9.4%	18.7%	15.9%	9.9%	15.2%	9.4%	12.9%	9.4%
	Percent mapped	78.3%	96.1%	97.3%	76.2%	76.2%	97.7%	81.5%	96.9%	97.4%	97.8%
Right reads:	Initial number of reads	87185432	57368915	15186094	1E+08	45937372	13847980	22022374	28236087	12437526	15940351
	Minimum per base quality score (10th percentile)	14	14	27	14	14	27	27	27	27	27
	Percent filtered in (%)	81.38	80.83	N/A	81.86	80.26	N/A	N/A	N/A	N/A	N/A
	Alignment input	70950705	46369117	15186094	81953371	36870545	13847980	22022374	28236087	12437526	15940351
	Mapped	56026587	44570183	14597762	62738333	28269426	13259768	17647947	26044316	11953398	15344925
	Multiple alignments	18.4%	19.2%	9.4%	18.7%	8.2%	9.9%	15.1%	9.3%	12.8%	9.4%
	Percent mapped	79.0%	96.1%	96.1%	76.2%	76.7%	95.8%	80.1%	92.2%	96.1%	96.3%

Table S2. Alignment statistics of *mdm* samples.

		EDL_R1	EDL_R2	EDL_R3	EDL_R4	EDL_R5	Psoas_R1	Psoas_R2	Psoas_R3	Psoas_R4	Psoas_R5	Soleus_R1	Soleus_R2	Soleus_R3
Age (days)		38	38	42	38	38	29	38	42	38	38	33	36	38
RNA integrity number (RIN)		8.6	8	7.9	7.8	8.1	7.7	7.8	7.8	7	7.5	8.7	7.5	7.8
Average Library size		262	323	265	262	262	258	287	277	262	262	290	279	262
	Initial number of reads	53885039	34932333	15793758	19829205	15148894	42440352	26748973	18199625	17724225	21623821	41514793	23439543	11885279
	Minimum average per base quality score	32	32	32	32	32	30	32	32	32	32	32	32	32
Left reads:	Percent filtered in (%)	NA	NA	77%	88%	NA	82%	NA	NA	NA	NA	NA	NA	87%
	Alignment input	53885039	34932333	12148686	17394262	15148894	34823448	26748973	18199625	17724225	21623821	41514793	23439543	10386271
	Mapped	49508738	33952263	11887172	16686560	14436007	32915940	25658557	17475681	17003352	20516046	39386741	22523933	9583779
	Multiple alignments	16.60%	8.50%	9.50%	32	32	9.00%	7.80%	6.80%	32	32	9.70%	7.50%	30
	Percent mapped	91.90%	97.20%	97.80%	95.93%	95.29%	94.50%	95.90%	96.00%	95.93%	94.88%	94.90%	96.10%	92.27%
	Initial number of reads	53885039	34932333	15793758	19829205	15148894	42440352	26748973	18199625	17724225	21623821	41514793	23439543	11885279
	Minimum average per base quality score	32	32	32	32	30	30	27	27	30	30	27	27	32
Right reads:	Percent filtered in (%)	NA	NA	77%	88%	NA	82%	NA	NA	NA	NA	NA	NA	87.39%
	Alignment input	53885039	34932333	12148686	17394262	15148894	34823448	26748973	18199625	17724225	21623821	41514793	23439543	10386271
	Mapped	48848914	32872092	11658223	16644030	13857045	32889036	24621394	17269780	16465048	20072739	38831363	22091290	9557932
	Multiple alignments	16.60%	8.40%	9.40%			8.40%	7.80%	6.80%			9.70%	7.50%	

	Percent mapped	91.70%	94.10%	96.00%	95.69%	91.47%	94.40%	92.00%	94.90%	92.90%	92.83%	93.50%	94.20%	92.00%
